# Strong Association between Diarrhea and Concentration of Enterotoxigenic *Escherichia coli* Strain TW10722 in Stools of Experimentally Infected Volunteers

**DOI:** 10.3390/pathogens12020283

**Published:** 2023-02-08

**Authors:** Oda Barth Vedøy, Hans Steinsland, Sunniva Todnem Sakkestad, Halvor Sommerfelt, Kurt Hanevik

**Affiliations:** 1 Department of Clinical Science, Faculty of Medicine, University of Bergen, 5021 Bergen, Norway; 2 Centre for Intervention Science in Maternal and Child Health (CISMAC), Centre for International Health, Department of Global Public Health and Primary Care, Faculty of Medicine, University of Bergen, 5020 Bergen, Norway; 3 Department of Biomedicine, Faculty of Medicine, University of Bergen, 5020 Bergen, Norway; 4 Cluster for Global Health, Division for Health Services, Norwegian Institute of Public Health, 0430 Oslo, Norway; 5 Norwegian National Advisory Unit on Tropical Infectious Diseases, Department of Medicine, Haukeland University Hospital, 5021 Bergen, Norway

**Keywords:** experimental infection in humans, diarrhea, enterotoxigenic *Escherichia coli*, heat-stable enterotoxin, controlled human infection model, feces, vaccine, quantitative PCR, small intestine

## Abstract

Enterotoxigenic *Escherichia coli* (ETEC) strains are a major cause of diarrheal illness in children and travelers in low- and middle-income countries. When volunteers are infected with ETEC strains, as part of experimental infection studies, some do not develop diarrhea. To improve our understanding of how these volunteers are protected, we investigated the association between stool ETEC DNA concentration, as determined by quantitative PCR, and the development and severity of disease in 21 volunteers who had been experimentally infected with ETEC strain TW10722. We found a strong association between maximum stool ETEC DNA concentration and the development of diarrhea: all of the 11 volunteers who did not develop diarrhea had <0.99% TW10722-specific DNA in their stools throughout the follow-up period of up to 9 days, while all of the 10 volunteers who did develop diarrhea had maximum DNA concentrations of ≥0.99%. Most likely, these maximum stool TW10722 DNA concentrations reflect the level of intestinal colonization and the risk of experiencing diarrhea, thereby, seems to be directly dependent on the level of colonization. Thus, the development and availability of vaccines and other prophylactic measures, even if they only partially reduce colonization, could be important in the effort to reduce the burden of ETEC diarrhea.

## 1. Background

Enterotoxigenic *Escherichia coli* (ETEC) comprise a group of pathogenic *E. coli* that, similar to other *E. coli*, are transmitted through the fecal–oral route, commonly through the ingestion of contaminated food and water [1]. They non-invasively colonize the epithelial cells lining the small intestine of the host and cause acute, self-limiting diarrhea [1]. ETEC are among the most important bacterial causes of diarrhea, responsible for a yearly estimate of around 220 million episodes globally [2]. The diarrheal burden caused by these diarrheagenic pathogens is largest among young children living in low- and middle-income countries (LMIC), where infections are endemic, and occur among travelers visiting these countries [2,3]. Efforts are ongoing to develop effective protective measures against ETEC [4,5,6].

During colonization, ETEC produce mucinases, which clear a path through the mucus layer that, when intact, prevents bacterial access to the small intestinal wall [7]. Most ETEC also produce one or more of several known colonization factors (CFs) that facilitate adherence to the epithelial cell surface [8]. The subsequent expression of heat-labile toxin (LT) and/or the heat-stable toxin (ST) induces diarrhea in the host [9]. ETEC have emerged from different parts of the *E. coli* population on several occasions and are, therefore, a phylogenetically diverse pathovar [10,11]. Some ETEC appear to be more pathogenic than others, as demonstrated by the variation in diarrheal attack risks and diarrheal severity following infection with different ETEC strains [12]. Furthermore, infections with ETEC that express certain colonization factors and the human variant of ST, STh, in particular, have been shown to be more strongly associated with the development of moderate and severe diarrhea than others [13,14,15]. 

Many volunteers experience no or only mild signs and symptoms when they are experimentally infected with ETEC, even when infected with the most pathogenic ST-producing ETEC strains [12,16]. Though the underlying mechanisms behind this apparent resistance to ETEC-induced disease are not yet fully understood, results from several studies suggest an association with suboptimal colonization [17,18,19,20,21]. For example, we previously found that volunteers who only excreted low levels of ETEC DNA after experimental infection with the ST-only ETEC strain TW11681 also had no relevant signs or symptoms. Further, they seemed to mount only low levels of strain-specific serum antibodies in response to the infection [21]. 

Finding the root causes of what appears to be a natural resistance to ETEC-induced diarrhea is important since it may help to identify new targets for developing preventive measures against ETEC. To contribute to this effort, here we investigated to what extent the level of colonization, as estimated using strain-specific qPCR on stool DNA, was associated with the development of disease in 21 volunteers who had been experimentally infected with the epidemiologically relevant ETEC strain TW10722 [22].

## 2. Methods

### 2.1. Strain Description

ETEC strain TW10722 (O115:H5; GenBank BioProject: PRJNA59745) was isolated in Guinea-Bissau in 1997 from the stool of a 15-month-old child who had acute diarrhea [15]. The strain does not produce LT but produces STh as well as the ETEC colonization factors of Coli Surface Antigen 5 (CS5) and 6 (CS6). We consider TW10722 to be an epidemiologically important strain, given that it belongs to the large ETEC5 or L5 ETEC lineage, which is commonly found to be associated with childhood diarrhea [10,11].

### 2.2. Experimental Infection Study

The study is based on clinical specimens and data collected during an experimental infection study conducted at the University of Bergen and Haukeland University Hospital, Bergen, Norway, between 2014 and 2018. A detailed description of that study, which was undertaken to develop a human challenge model for testing new ST-based ETEC vaccines, is found elsewhere [22]. In total, 21 volunteers were experimentally infected with TW10722. The volunteers included 18 females and 3 males with ages ranging from 19 to 29, who had no travel history to resource-limited areas during the 12 months prior to the start of the study. The volunteers were offered regular meals during their stay at the hospital, but they were not required to adhere to any dietary restrictions during follow-up.

The volunteers were enrolled in the study in groups of three, and each group shared a cohort isolation room at the hospital for the duration of the follow-up period. Prior to ingesting the dose, the volunteers fasted for 11 h before drinking 130 mL of 1.33% bicarbonate buffer (158 mM NaHCO_3_), followed one minute later by orally ingesting dosages of 1 × 10^6^ (n = 3), 1 × 10^7^ (n = 3), 1 × 10^8^ (n = 3), 1 × 10^9^ (n = 3), or 1 × 10^10^ (n = 9) CFUs of ETEC strain TW10722 suspended in 30 mL of the bicarbonate buffer. Fasting ended one hour after ingesting the dose. The volunteers were subsequently admitted to an infection isolation ward and followed with daily stool specimen collection, clinical examinations, and self-reported clinical signs and symptoms. Blood specimens were collected on the day of dose ingestion and 10 and 28 days afterward. Stools were immediately stored at 4 °C for a maximum of 28 h before homogenization by stirring and storage at −70 °C. For each volunteer, we stored and analyzed the specimens from up to three evacuations for each follow-up day. 

A test for the excretion of viable ETEC was performed on each volunteer at least once each day during follow-up to monitor the infection. A collected stool specimen, or a rectal swab specimen if no stools were available, was streaked onto Lactose agar, and after overnight incubation, a swipe of the colonies was tested for the presence of the STh gene (*sta3*; UniProt ID: P07965) using qPCR, as described by Skrede et al. [23]. The infection was cleared by a 3-day ciprofloxacin treatment that started, at the latest, five days after dose ingestion (study day 5). The treatment was started earlier if the volunteers developed moderate or severe diarrhea (defined below) lasting for ≥24 h, if they developed mild diarrhea in addition to having had two or more other symptoms (among fever, vomiting, abdominal pain/cramps, headache, myalgias and nausea) for at least two days, or if the senior physician considered it necessary. Volunteers were discharged from the isolation ward, and no further stool specimens were collected when the volunteers had started ciprofloxacin treatment and had provided 3 successive stool specimens in which no viable ETEC could be detected.

### 2.3. Disease Characterization

Each stool specimen was graded from 1 to 5 based on whether it was firm and formed (Grade 1), soft and formed (Grade 2), viscous, opaque liquid or semiliquid (Grade 3), opaque liquid (Grade 4), or clear or translucent liquid (Grade 5). Stools of grades 3, 4, or 5 were defined as being loose. The volunteers were considered to have diarrhea when they passed 1 loose/liquid stool (grade ≥3) totaling ≥300 g or ≥2 loose/liquid stools totaling ≥200 g during any 48-h period within 120 h after dose ingestion. A diarrheal episode was defined as the period between the first to the last passed diarrheal stool, also allowing for grade 1 or grade 2 stools to be passed during this period. A diarrheal episode was defined as being mild if the volunteer experienced up to 3 loose stools and/or a total stool weight of ≤400 g, moderate if the volunteer experienced 4–5 loose stools and/or a total stool weight of 401–800 g, and severe if the volunteer experienced ≥6 loose stools and/or a total stool weight of ≥801 g during any 24-h period of the diarrheal episode [24]. 

For each volunteer, a disease severity score ranging from 0 (least severe) to 8 (most severe) was estimated based on a combined scoring of objective signs, subjective symptoms, and diarrhea severity, as described by Porter et al. [22,25]. This included the following: several bouts of vomiting during any 24-h period and/or any fever (score = 2), 1 bout of vomiting during any 24-h period without any fever (score = 1), any episodes of moderate to severe lightheadedness and/or severe nausea, malaise, headache or abdominal cramp (score = 2), mild lightheadedness and/or mild to moderate nausea, malaise, headache or abdominal cramps (score = 1), during any 24-h period within a diarrheal episode: >1000 g and/or >12 stool evacuations (score = 4), >600 to ≤1000 g and/or >7 to ≤12 evacuations (score = 3), >400 to ≤600 g and/or >4 to ≤7 evacuations (score = 2), and >0 to ≤400 g and/or >1 to ≤4 evacuations (score = 1).

### 2.4. Stool TW10722 DNA Concentration Estimation

To measure TW10722 DNA concentrations in the stools of these volunteers, we isolated the total DNA from their specimens and used a TW10722-specific qPCR assay to estimate the percentage of DNA originating from TW10722. The stool DNA was isolated from around 0.2 g of a thawed stool specimen using an in-house-developed, manual, high-throughput purification method, as described previously [21]. The purified DNA was resuspended in a dilution buffer (10 mM Tris-HCl; pH 8.0; 0.05% Tween-20) and stored at −70 °C until use. 

To quantitate TW10722 DNA, we developed a probe-based qPCR assay that specifically targets the *E. coli* O115-specific variant of the O-antigen polymerase gene (*wzy*), which is unique to serogroup O115 *E. coli* [26], and only present at a single chromosomal site in TW10722. By comparing the cycle threshold values between qPCR performed on purified stool DNA with those from a dilution of known amounts of TW10722 DNA, it is possible to estimate the amount of TW10722 DNA present in the qPCR assay. By dividing these results by the total amount of DNA used as a template in each qPCR assay, we estimated the relative DNA concentration, in %, that originated from TW10722. The forward (CGATGATGTTGCTATTACTAC; O115_wzy_TF) and reverse (GAACTACTACCAGAGGATTC; O115_wzy_TR) primers were used to amplify a 137 bp section of *wzy* from nucleotide 717 to 853, and the probe (TCACCGCTTGCCTAAATGGTTCT; O115_wzy_TP), which binds the forward strand at nucleotide 771, was labeled with 6-FAM (5′-end) and Onyx Quencher A (3′-end).

Immediately before performing the qPCR, the thawed stool DNA was quantified using the QuantiFluor dsDNA System (Promega Corporation, Madison, WI, USA) and subsequently diluted in a dilution buffer to final concentrations of 1.0 ng/µL and 0.1 ng/µL. Each 9 µL reaction contained 1× ABsolute qPCR mix (Thermo Fisher Scientific, Waltham, MA, USA), 0.4 µM of both O115-wzy-TF and O115-wzy-TR primers, 0.2 µM O115-wzy-TP probe, and 1.5 µL of diluted stool DNA as a template. Each DNA extract from the stool specimens was analyzed in four replicates, two for each of the above-mentioned template dilutions, in a 384-well PCR plate on a LightCycler 480 machine (Roche, Basel, Switzerland). The plates were incubated for 15 min at 95 °C, followed by 45 cycles of 20 s at 95 °C and 90 s at 60 °C. 

As positive controls, we used purified TW10722 DNA mixed with equal quantities of DNA from two non-O115 *E. coli*, including ETEC strain TW11681 and *E. coli* BL21 (DE3). This DNA was diluted so that it contained 0.5 ng/µL of TW10722 DNA, and a 10-fold dilution series was prepared from 0.5 ng/µL to 50 fg/µL and a two-fold series to 25 and 12.5 fg/µL. These were used as templates in the triplicate reactions, while the dilution buffer was used as a template for the negative control (also in triplicate). Given that the genome of a single TW10722 bacterium consists of around 5.6 × 10^6^ base pairs and consequently weighs around 6.0 fg, the positive control dilution series reactions contained from around 125,000 (for the 0.5 ng/µL dilution) to 3 (for the 12.5 fg/µL dilution) copies of the TW10722 genome.

The quantitation cycle for each qPCR curve was estimated using the Second Derivative Maximum Method in the LightCycler 480 Software, version 1.5.1.62 (Roche Life Science). The same software was also used to generate the calibration curve for the positive control dilution series and to estimate PCR efficiency.

### 2.5. Immunological Assays

To evaluate whether existing or induced anti-TW10722 immunity could explain some of the variations in stool TW10722 DNA concentrations, we investigated the association between stool TW10722 DNA concentrations and the levels of serum antibodies that target important TW10722 virulence factors. The immunological data have, in part, already been reported by Sakkestad et al. [27] and were generated using a multiplex bead-based flow cytometric immunoassay, where the beads were coupled with recombinantly produced and purified CS5 or YghJ. YghJ (*yghJ*; UniProtKB ID: P33781) is a conserved mucin-degrading *E. coli* metalloprotease [7], and CS5 (*csfA;* UniProtKB ID: P0CK95) is a colonization factor involved in anchoring ETEC to the intestinal epithelial cells [8]. While CS5 is most likely only produced by ETEC belonging to the ETEC5/L5 lineage [10,11], YghJ is produced by most pathogenic *E. coli* [28]. Therefore, most of our volunteers were likely to have little or no previous exposure and pre-existing immunity to CS5 but a relatively frequent exposure and stronger pre-existing immunity to YghJ. 

### 2.6. Statistical Analysis

All of the analyses and figures were made in R, version 4.1.1 [29]. Wilcoxon Rank Sum tests were used to estimate the association between the maximum observed stool TW10722 DNA concentration and the development of diarrhea. Linear regression analyses were used to estimate the association between the maximum observed stool TW10722 DNA concentration and the severity of the diarrheal episode and between the maximum observed stool TW10722 DNA concentration and the disease severity score.

## 3. Results

In this study, we used qPCR to estimate the relative amount of TW10722 genomic DNA in daily collected stools from 21 experimentally infected volunteers and investigated the association between these DNA concentrations and the development of disease as well as pre-existing and induced serum antibody responses targeting TW10722 antigens.

### 3.1. Follow-Up

**Figure 1:**
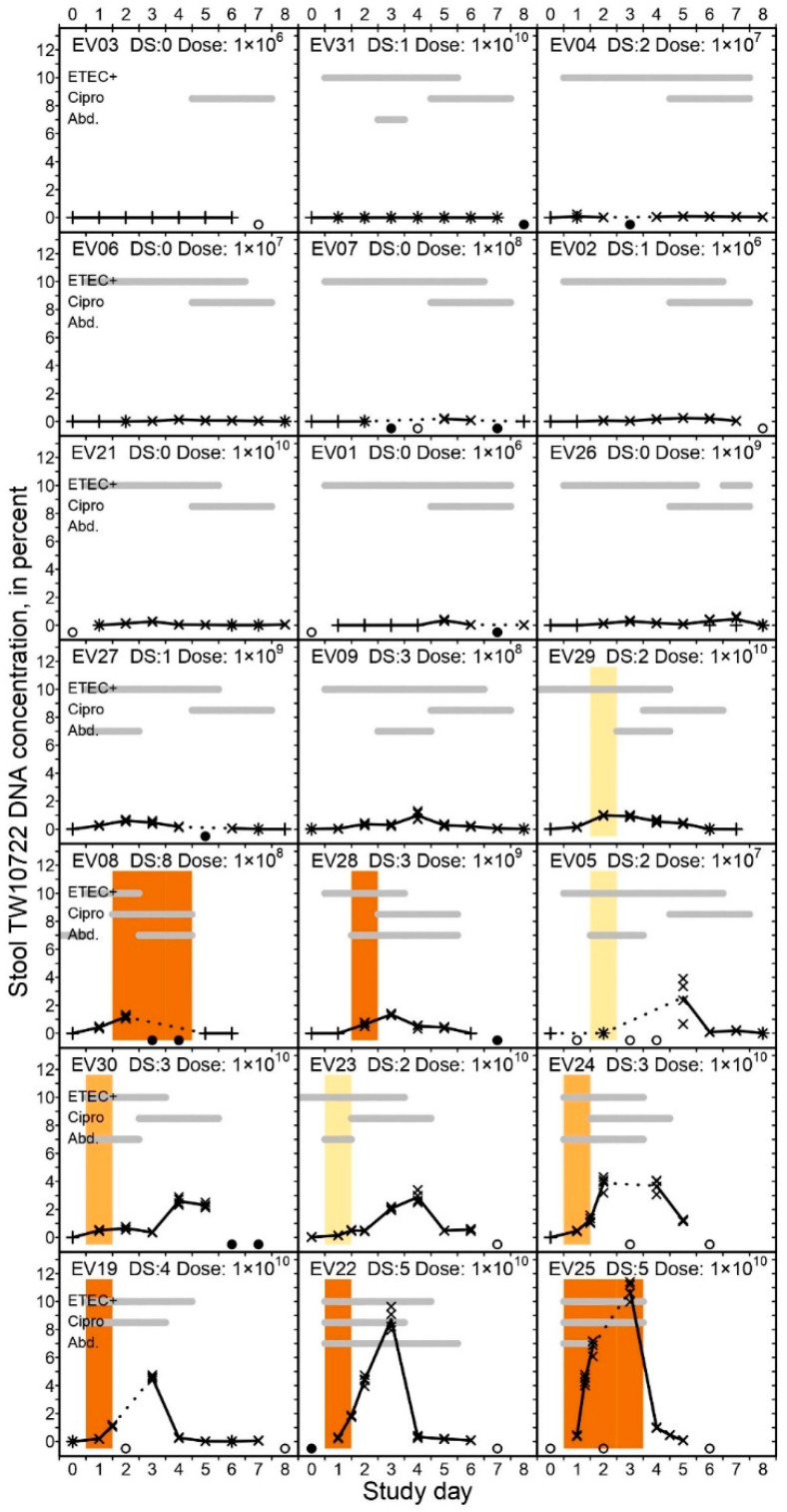
Percent TW10722 DNA concentration in stools of experimentally infected volunteers. The volunteers are sorted ascendingly on maximum TW10722 DNA concentrations seen during the 9-day follow-up period. The scatter plots show the estimated TW10722 DNA concentrations based on individual qPCR results, with crosses (×) and plusses (+) indicating whether the estimate fell inside or outside the linear dynamic range of the assay, respectively. The plot lines are connected through the mean estimated TW10722 DNA concentration based on qPCR results for the 4 technical replicates performed on each analyzed stool specimen. Dotted lines are used to indicate days when qPCR results are missing, with open (○) or closed (●) circles at the bottom of each graph indicating whether the omission was due to no stools being shed on the given day, or the stool was not analyzed, respectively. For days when specimens from >1 stool evacuation were available, we evenly distributed the results across the given day on the x-axis. In each graph, the gray horizontal bars indicate on which days the volunteers had ETEC-positive stools or rectal swabs (“ETEC+”), received antibiotic treatment (“Cipro”), or had abdominal pains or cramps (“Abd.”). The heading in each plot indicates the Volunteer ID number, the disease severity score (“DS:”) and the dose ingested (“Dose:”) in CFU. The colored vertical bars span the period when the given volunteer experienced a diarrheal episode, and the color of the bar indicates the severity of the episode, with light yellow indicating mild diarrhea, light orange indicating moderate diarrhea, and dark orange indicating severe diarrhea.

Of the 21 volunteers, twelve (57%) were followed for all 9 follow-up days, six (29%) were followed for 8 days, and three (14%) were followed for 7 days, totaling 177 days of follow-up. Viable ETEC were detected in the stool and rectal swab specimens of 94 (53%) of the 177 volunteer-days, with only EV03 having no viable ETEC detected in her stools throughout the follow-up period. For all of the other volunteers, the median last day of detection was on study day 5 (IQR: 3–6; range: 2–7) (Figure 1). The lack of viable ETEC after study day 7 was expected due to the fact that the volunteers started ciprofloxacin treatment on study day 5, at the latest. 

### 3.2. Signs and Symptoms

As first reported by Sakkestad et al. [22], of the 21 volunteers, ten (48%) developed diarrhea, including three (14%) who developed mild diarrhea, two (10%), who developed moderate diarrhea, and five (24%) who developed severe diarrhea. These episodes lasted for a median of 9 (interquartile range [IQR]: 4–13; range: 0–66) hours. Other self-reported signs and symptoms that were experienced by the volunteers during the follow-up period included abdominal pain (n = 11; 52%), abdominal cramping (n = 11; 52%), bloating (n = 11; 52%), nausea (n = 10; 48%), malaise (n = 10; 48%), headache (n = 9; 43%), excessive flatus (n = 7; 33%), lightheadedness (n = 5; 24%), decreased appetite (n = 3; 14%), vomiting (n = 2; 10%), fever (n = 2; 10%), constipation (n = 1; 5%), chills (n = 1; 5%), and generalized myalgias (n = 1; 5%). Finally, six of the twenty-one volunteers had a disease severity score of zero, three had 1, four had 2, four had 3, one had 4, two had 5, and one had a score of 8, which is the maximum possible severity score.

### 3.3. Stool Specimen Collection and Characterization

Of the 177 follow-up days, no stools were passed on 17 (9.6%) of the days, no useful amount of DNA could be extracted from the specimens collected on 7 (4.0%) of the days, and the specimens were not properly stored on 5 (2.8%) of the days. Consequently, the stool specimens were successfully analyzed for 148 (84%) of the 177 volunteer-days. Due to the fact that specimens from up to three stool evacuations for each volunteer-day were included in our analyses, 142 (96%) of the 148 volunteer-days were represented by specimens from a single evacuation, 5 (3.4%) were from two evacuations, and 1 (0.7%) was represented by specimens from three evacuations from the same volunteer. In total, 155 stool specimens were analyzed and included in this study.

Of the 155 included specimens, twelve (7.7%) were collected during a diarrheal episode, of which six were from grade 3 and six were from grade 4 stools. Of the seven specimens we failed to analyze, two (29%) were from a diarrheal episode and of grades 3 and 4. The median total stool output during a diarrheal episode was 516 (IQR: 389–551; range: 286–2754) grams. Outside of a diarrheal episode, the median, mean daily stool outputs between volunteers who did and did not experience diarrhea were similar (100 [IQR: 72–154] grams vs. 131 [IQR: 108–154] grams, respectively; Wilcoxon rank sum test: *p* = 0.25). Including diarrheal episodes, the corresponding daily stool outputs were 253 [IQR: 223–279] vs. 131 [IQR: 108–154] grams, respectively; *p* < 0.001).

### 3.4. TW10722 DNA Quantitation Assay Validation

Based on the calibration curves generated from the positive control dilution series, the efficiency of our qPCR analyses ranged between 96% and 104%. The assay had a linear dynamic range for detecting between 3 and 125,000 TW10722 genomes, and the results that fell outside of the lower end of this linear dynamic range were considered negative. No results fell outside of the higher end of the linear dynamic range, and none of the negative controls showed signs of amplification.

The qPCR results did not appear to be affected by the PCR inhibitors in the template DNA. For each of the 155 specimens included in the study, we quantified the TW10722 DNA in four replicates, including two based on 0.1 ng/µL and two based on 1.0 ng/µL template DNA. If PCR inhibitors were present in the template DNA and affected the PCR efficiency, we would expect that the quantitation cycle (Cq) differences between the PCR based on the two template concentrations would be skewed towards less than 3.32 cycles. Instead, we found that the median Cq difference was 3.49 and that the differences were normally distributed (Shapiro–Wilk Normality Test *p*-value, 0.64; skewness coefficient, 0.04). The only outlier in these analyses was the EV21-Day 1 specimen, which had a mean Cq difference of 1.83. For this specimen, the 0.1 ng/µL template reactions were close to the lower detection limit of our assay, with only one of the two replicates detecting TW10722 DNA. Therefore, we expect that the abnormally low Cq difference for this specimen was a result of an inaccurate Cq estimate rather than a result of PCR inhibition.

When testing the stool specimens collected later the same day that strain TW10722 was ingested (study day 0), we found no clear indication that the assay had a low specificity or that the volunteers already harbored other O115-positive microorganisms at the start of the study, which could otherwise have biased our quantitation assay results. Of the 17 specimens collected on study day 0, fourteen (82%) were assay-negative, while the remaining three (18%) specimens were weakly positive, containing <0.02% TW10722 DNA. This most likely represented TW10722 bacteria that quickly passed through the gut as a result of fasting.

### 3.5. TW10722 Shedding and Diarrhea

All of the volunteers had TW10722 DNA detected in their stools during follow-up, except for EV03, who did not excrete any detectable amount of TW10722 DNA. TW10722 DNA was detected in 120 (77%) of the 155 stool specimens included in the study, with the median last day of detection being on study day 7 (IQR: 5–8; range: 2–8). For the 11 volunteers who did not develop diarrhea, the stool TW10722 DNA concentration remained low throughout the follow-up period (Figure 1), with a median maximum concentration of 0.25% (IQR: 0.11–0.40%; range: 0.00–0.98%). For the 10 volunteers who did develop diarrhea, the maximum stool TW10722 DNA concentrations were clearly higher, peaking at a median of 2.74% (IQR: 1.66–4.40%; range 0.99–10.79%, Wilcoxon rank sum test, *p* < 0.001). The maximum concentrations were seen on median study day 4 (range 2–7) for the 11 volunteers who did not develop diarrhea. For the volunteers who experienced diarrhea, the stool TW10722 DNA concentration peaked on median day 3 (range 2–5).

Among the volunteers who developed diarrhea, the median TW10722 DNA concentration in the last non-diarrheal stool was somewhat lower than the concentration in the subsequent first diarrheal stool (0.14% [IQR: 0.00–0.34%] vs. 0.64% [IQR: 0.50–1.22]; Wilcoxon rank sum test, *p* = 0.009). This was expected, as the last non-diarrheal stool often was a Day 0 stool, which was passed before ETEC was expected to have colonized and passed through the gut. However, the TW10722 DNA concentrations were similar when comparing the last diarrheal stool with the first consecutive non-diarrheal stool among these volunteers (median 1.16% [IQR: 0.61–1.41%] vs. median 1.17% [IQR: 0.61–4.00%]; Wilcoxon rank sum test, *p* = 0.96).

### 3.6. TW10722 Shedding and Disease Severity

**Figure 2:**
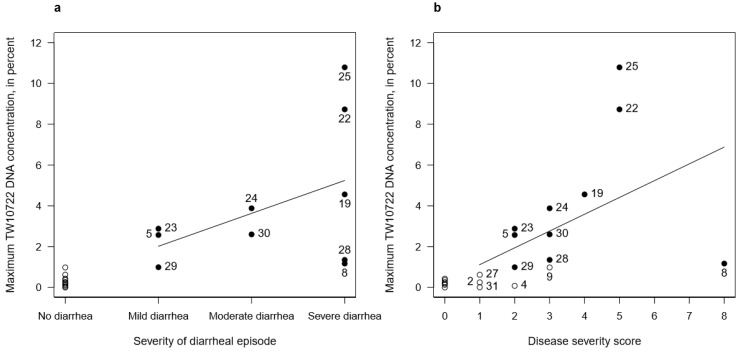
Association between maximum stool TW10722 DNA concentrations and disease severity estimates, including the severity of the diarrheal episode (**a**) and disease severity score (**b**). Closed circles (●) represent the 10 volunteers who developed diarrhea, while open circles (○) represent the 11 volunteers who did not. The EV identity numbers are shown for volunteers who developed diarrhea (**a**) and for volunteers who had disease severity scores ≥1 (**b**). Solid lines represent the regression lines for the association between severity and TW10722 DNA concentrations.

Given the clear association between the maximum observed stool TW10722 DNA concentration and experiencing diarrhea, we evaluated whether the maximum DNA concentrations seen in these volunteers could correlate with the severity of the diarrheal episode and with disease severity. Using linear regression on the data taken from the 10 volunteers who developed diarrhea, we found that the maximum stool TW10722 DNA concentration increased by 1.61% (95% CI: −1.01–4.23%) for each step increase in the severity of the diarrheal episode (i.e., from mild to moderate, and from moderate to severe diarrhea; Figure 2a). The disease severity score incorporates, in addition to diarrhea severity, other signs and symptoms experienced by the volunteers. Performing these analyses for the 15 volunteers who had disease severity scores ≥1, the maximum stool TW10722 DNA concentration increased by 0.82% (95% CI: −0.05–1.70%) for each point increase in disease severity score (Figure 2b). Performing these same analyses for all of the 21 volunteers, the corresponding estimates were 1.65% (95% CI: 0.90–2.39%) and 0.83% (95% CI: 0.30–1.36%), respectively. 

### 3.7. TW10722 Shedding and Antibody Responses 

To evaluate whether the variation in maximum stool TW10722 DNA concentrations could be explained by pre-existing or acquired immunity to TW10722, we assessed the associations between the levels of existing or acquired serum antibodies targeting the TW10722 virulence factors CS5 or YghJ and maximum stool TW10722 DNA concentration in each volunteer.

**Figure 3:**
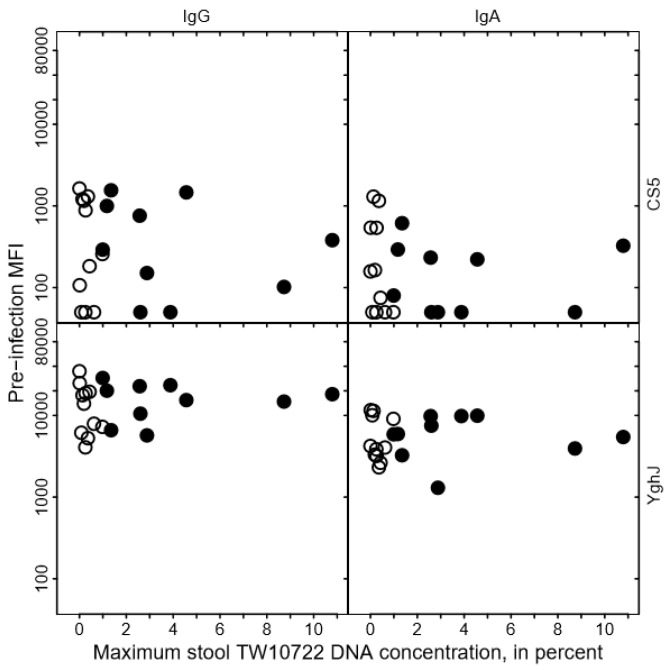
Relationship between CS5- and YghJ-specific serum IgA and IgG antibody levels prior to TW10722 infection and maximum stool TW10722 DNA concentration, in percent. Pre-infection MFI represents the median fluorescence intensity (MFI) of the bead-based flow-cytometry assay. Closed circles (●) represent the 10 volunteers who developed diarrhea, and open circles (○) represent the 11 volunteers who did not. For volunteers who had MFI values of <50, the MFI values were set to 50.

**Figure 4:**
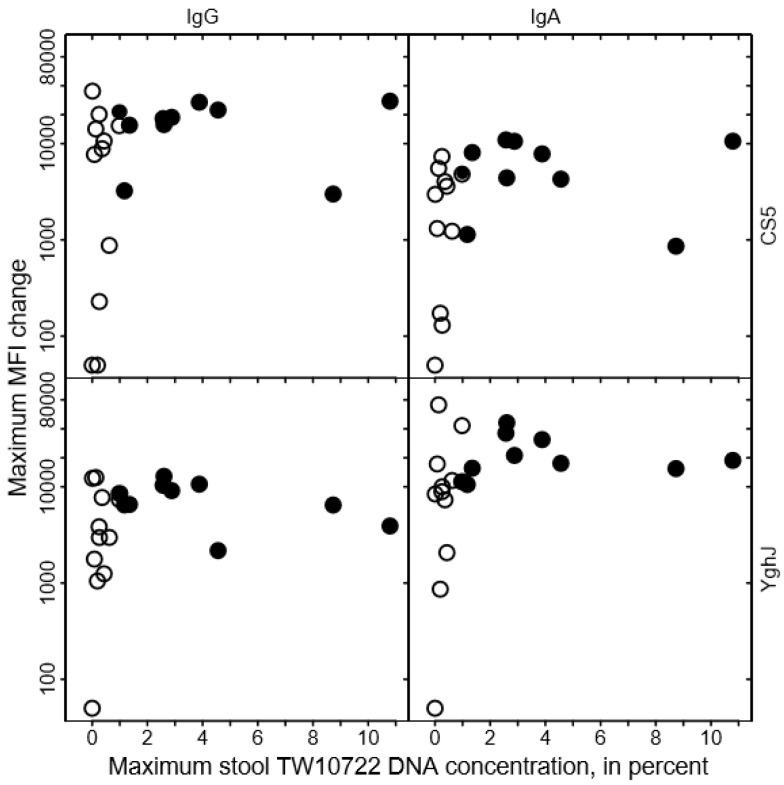
Relationship between changes in CS5- and YghJ-specific serum IgA and IgG levels following TW10722 infection and maximum stool TW10722 DNA concentration, in percent. Maximum MFI change represents the difference in the median fluorescence intensity (MFI) of the bead-based flow-cytometry assay between specimens taken on the day of ingesting the dose and on the day, either study day 10 or 28, that the highest MFI values were observed. Closed circles (●) represent the 10 volunteers who developed diarrhea, while open circles (○) represent the 11 volunteers who did not. For volunteers who had a difference in MFI of <50, the maximum MFI change was set to 50.

We found no clear association between the levels of anti-CS5 and anti-YghJ serum IgA or IgG and maximum stool TW10722 DNA concentrations for these volunteers, either before infection (Figure 3) or after the infection was cleared (Figure 4). Some of the volunteers who had low maximum TW10722 DNA concentrations and who did not develop diarrhea also seemed to have relatively low increases in anti-CS5 and YghJ antibody levels (Figure 4). Furthermore, pre-existing levels of serum antibodies targeting the TW10722 virulence factors CS5 and YghJ were similar among the volunteers who developed and who did not develop diarrhea (Figure 3).

## 4. Discussion

In this study, we evaluated the association between stool ETEC DNA concentrations and the development of disease in 21 volunteers who had been experimentally infected with the ETEC strain TW10722. We found that the association between the maximum observed stool TW10722 DNA concentration and experiencing diarrhea was clear-cut: all of the volunteers who had ≥0.99% TW10722 DNA in their stools at some time during their follow-up period experienced diarrhea, whereas those who always had <0.99% did not. Furthermore, this maximum TW10722 DNA concentration also seemed to increase with the severity of the diarrheal episode and with disease severity.

Given that we report TW10722 DNA concentrations relative to the total stool DNA concentrations, it is expected that some of the variations in our estimates may be attributed to variations in the concentration of fecal DNA contributed by other microbial and human cells. Several factors or events could potentially have influenced the total DNA concentration [30]. For example, it is possible that the diarrheal episode could have flushed out other cells from the colon or dislodged comparably more TW10722 cells from the small intestinal cell wall, both of which could have affected the estimated TW10722 DNA concentration. However, we found that the effects of the diarrheal episode probably did not lead to artificially high TW10722 DNA concentration estimates. For example, we found no clear indication that diarrheal stools, in general, had higher TW10722 DNA concentrations than their closest subsequent non-diarrheal stools. Furthermore, of the eight volunteers who had stools from diarrheal episodes analyzed, only two (25%) had maximum stool TW10722 DNA concentrations represented by diarrheal stools.

Similarly, it seems likely that any large variation in dietary intake could also have affected total microbial and human DNA levels in the stools [30]. Thus, if the volunteers that developed diarrhea lost their appetite and had a lower dietary intake, this could also have influenced the estimated TW10722 DNA concentration levels. However, the volunteers’ median daily weight of stools outside any diarrheal episode appeared to be similar for those who did and those who did not develop diarrhea, suggesting that the observed TW10722 DNA concentrations were probably not substantially influenced by differences in the dietary intake between those who did and those who did not develop diarrhea.

The most likely explanation for the clear-cut association between the maximum stool TW10722 DNA concentration and experiencing diarrhea is that stool TW10722 DNA concentration reflects the level of colonization in the small intestine and that the risk of developing diarrhea for these volunteers increased with increasing levels of colonization. Although the immune system and other host-dependent protective factors probably play an important role in limiting the risk of colonization and diarrhea with ETEC, we found no clear indication that pre-existing levels of anti-TW10722 serum antibodies were associated with either colonization level or with the development of diarrhea. On the other hand, for some volunteers, low colonization levels did seem to be associated with poor antibody responses, suggesting that the level of colonization could also influence the strength of the antibody responses against TW10722.

Pop et al. [31] also reported an association between developing diarrhea and having large concentrations of *Escherichia* spp. in stools following experimental infection with ETEC strain H10407, which produces LT, STh, and STp toxins. Using 16S rRNA sequencing, they found that the proportion of *Escherichia* spp. (most likely H10407) to other bacterial species ranged from 9% to 76% in the stools of volunteers who developed diarrhea and stayed below 1% in the stools of those who remained healthy [31]. While the use of different methodologies complicates direct comparisons between these two studies, the results suggest that the risk of developing diarrhea due to infection with H10407 could also be directly dependent on the level of colonization. 

In a previous experimental infection study using ETEC strain TW11681, the association between stool ETEC concentration and diarrhea was less clear-cut [21]. In that study, only two of the nine volunteers developed diarrhea (both mild episodes), and while both had relatively high maximum TW11681 DNA concentrations (3.7% and 8.2%), four of the seven volunteers who did not develop diarrhea also had comparably high concentrations (range: 3.3–5.7%). This suggests that for TW11681, colonization level may not be as a uniquely important risk factor for developing diarrhea as for TW10722 and H10407. Nevertheless, these are small studies performed in different populations under different conditions; therefore, larger studies are needed to confirm this variation. 

## 5. Conclusions

In this study, we found that the development of diarrhea in volunteers who were experimentally infected with ETEC strain TW10722 was clearly associated with the maximum observed TW10722 DNA concentration in the volunteers’ stools. This finding suggests that the maximum DNA concentration reflects the level of colonization in these volunteers and that it is the level of colonization that largely determines the risk of diarrhea and possibly disease severity. Identifying the underlying mechanisms behind why some of these volunteers appeared to be resistant to colonization could help identify new targets and strategies for preventing or treating ETEC infection and disease. If reducing the levels of ETEC colonization does protect against diarrhea from infection with the most pathogenic types of human ETEC, this offers hope that even the development of ETEC vaccines and other prophylactics that only partially protect against ETEC colonization could contribute to reducing the burden of ETEC diarrhea. 

## Data Availability

The data generated and analyzed in this study are available in the Supplementary Materials.

## Supplementary Materials

The following supporting information can be downloaded at: https://www.mdpi.com/article/10.3390/pathogens12020283/s1. The data presented in this study are available in the supplementary Excel workbook (Data Material).
